# The Impact of Fluid Balance on Clinical Outcomes in ICU Patients with Pulmonary Arterial Hypertension: A Retrospective Analysis using MIMIC-IV

**DOI:** 10.2174/0118743064418020251204090156

**Published:** 2026-01-26

**Authors:** Geran Maule, John Rickards, Abdallah Rayyan, Moh’d Alamin Daise, Kobina Essilfie Quaye, Peters Okonoboh

**Affiliations:** 1 College of Medicine, University of Central Florida, Orlando, FL, United States; 2 HCA Florida, North Florida Hospital, Graduate Medical Education Internal Medicine Residency Program, Gainesville, FL, United States; 3 Department of Internal Medicine, Mercer University School of Medicine, Macon, GA, United States

**Keywords:** Intensive care unit, Pulmonary arterial hypertension, MIMIC-IV, Right ventricular failure, Hemodynamic monitoring, PAC-Man trial

## Abstract

**Introduction:**

Pulmonary Arterial Hypertension (PAH) is a significant comorbidity that can complicate the care of patients in the Intensive Care Unit (ICU). PAH's hemodynamic burden and systemic effects add complexity to managing fluid balance, ventilatory support, and other critical care interventions. This study aims to investigate the impact of fluid balance in ICU patients with PAH.

**Methods:**

We conducted a retrospective cohort study using the MIMIC-IV v3.1 database. Patients with diagnosed PAH (ICD-9: 4160; ICD-10: I270) and complete ICU records were included. Exclusions were applied for missing data, duplicate ICU stays, and secondary causes of pulmonary hypertension. An initial cohort of 178 patients was identified. Further data validation led to a final cohort comprising 102 patients. Analyses were conducted in Google BigQuery and Python-based tools (Google Colab).

**Results:**

Among 102 patients, 77 (75.4%) had a positive fluid balance, and 25 (24.5%) had a negative balance. Positive fluid balance was associated with higher ICU mortality (14.49% *vs*. 5.88%), shorter ICU stays (2.29 *vs*. 3.06 days), and a 74% increase in ICU mortality odds (OR: 1.74, 95% CI: 1.10–2.85). The lowest ICU mortality was seen in patients with net fluid balance between -2000 mL and +2000 mL. Severely positive balance (>+5000 mL) was associated with the highest ICU mortality (19%) and longest ICU LOS (6.16 days).

**Discussion:**

This study highlights the critical importance of fluid balance in PAH patients, a population uniquely susceptible to right ventricular failure. The findings suggest that even modest positive fluid balance may worsen outcomes, supporting prior physiologic models linking volume overload to RV dysfunction. While limited by retrospective design and lack of hemodynamic data, the results underscore the potential benefit of individualized fluid strategies and the selective use of advanced monitoring. Further prospective research is needed to define safe fluid thresholds and guide therapy in this high-risk group.

**Conclusion:**

Our findings suggest that both excessive positive and severe negative fluid balance may be detrimental in ICU patients with PAH. A moderate fluid balance range (-2000 mL to +2000 mL) showed the most favorable outcomes.

## 1 INTRODUCTION

Pulmonary arterial hypertension (PAH) is a rare, highly complex and progressive disorder which if not managed appropriately can lead to premature death, especially for patients admitted in the intensive care unit (ICU) [1, 2]. It is characterized by a sustained increase in pulmonary vascular resistance (PVR) and right ventricular (RV) dysfunction often leading to eventual right heart failure and premature death [3, 4]. Despite advances in targeted therapies, PAH remains a condition with high morbidity and mortality, particularly in patients experiencing acute RV decompensation [5, 6]. Critically ill PAH patients face particularly grim outcomes, with reports indicating that up to 38% either die or require urgent transplantation within 90 days [7]. Fluid balance plays a pivotal role in hemodynamic stability and clinical outcomes in this population [8]. While studies in stable patients with PAH have linked high plasma volumes to worse outcomes, very few clinical studies exist assessing volume status in critically ill patients with pulmonary vascular dysfunction [8, 9]. Excessive positive fluid balance contributes to RV overload, increased right atrial pressure and pulmonary congestion, worsening cardiac output and organ perfusion [8, 10]. Conversely, aggressive fluid removal may induce hypoperfusion and systemic hypotension, compounding the risk of multiorgan failure [3, 11, 12]. These competing risks emphasize the delicate balance between maintaining adequate perfusion and avoiding volume overload when managing patients with PAH in the ICU [13-15].

Despite the critical role of fluid management in PAH outcomes, research on this topic remains limited [8]. Fluid management has been studied in ICU patients with sepsis, trauma, and cardiogenic shock, but little is known about its impact in pulmonary arterial hypertension [16]. Recent advances in machine learning, including ensemble learning methods for cardiovascular risk prediction, highlight the potential to develop improved prognostic tools for critical care populations. To our knowledge, no prior study has evaluated fluid balance specifically in ICU patients with PAH. This study aims to analyze the impact of fluid balance on ICU outcomes in PAH patients using data from the MIMIC-IV database. Specifically, we investigate the relationship between fluid balance and key clinical outcomes, including mortality, ventilator use, renal replacement therapy (RRT) use, and ICU length of stay (LOS). By identifying patterns in fluid management and their effects on PAH prognosis, we hope to provide valuable insights to optimize ICU treatment strategies in this high-risk population.

## 2 METHODS

### 2.1 Data Source

This study utilized the publicly available MIMIC-IV v3.1 database, a large, de-identified dataset containing clinical data of patients admitted to critical care units at Beth Israel Deaconess Medical Center between 2008 and 2019. (Johnson AEW, Stone DJ, Celi LA, Pollard TJ. The MIMIC-IV database: building a resource for critical care research. *Physiol Meas*. 2021).

### 2.2 Study Design

A retrospective cohort study was conducted to evaluate patients with PAH admitted to the intensive care unit (ICU). Data extraction and analysis were performed using Google BigQuery and Python-based tools (Google Colab).

### 2.3 Inclusion Criteria

Patients were included in the study if they met the following criteria:

(1) At least one ICU admission was recorded in the MIMIC-IV database.

(2) Documented diagnosis: *Idiopathic Pulmonary Arterial Hypertension (IPAH)*, identified using the following ICD codes:

o ICD-9: 4160

o ICD-10: I270

(1) Availability of ICU admission and discharge timestamps (*i.e*., intime and outtime per the MIMIC-IV database).

### 2.4 Exclusion Criteria

Patients were excluded if:

(1) Multiple admissions were recorded for the same patient without a unique identifier or resolution.

(2) Missing essential variables such as fluid balance, ventilator data, or demographic information.

(3) Diagnoses indicative of secondary pulmonary hypertension, such as left heart disease, chronic lung disease, and thromboembolic PH, were excluded based on ICD-9 and ICD-10 coding criteria. We focused only on idiopathic pulmonary arterial hypertension (IPAH), referred to herein as PAH.

### 2.5 Data Extraction

Using SQL queries in Google BigQuery, the cohort was identified by filtering ICU admissions linked to the specified ICD codes. Additional data on physiological parameters, fluid balance, ventilator use, ICU mortality, and diagnoses were extracted from the relevant tables in MIMIC-IV.

**Fig. (1):**
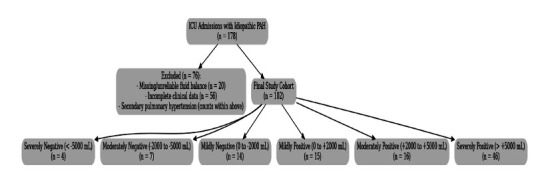
STROBE-compliant patient selection flowchart. A total of 178 ICU admissions with idiopathic pulmonary arterial hypertension (IPAH) were initially identified from the MIMIC-IV database. After excluding 20 patients with missing or unreliable fluid balance data and 55 patients with incomplete clinical records (including cases of secondary pulmonary hypertension), 102 patients remained in the final study cohort. These patients were stratified into six fluid balance subgroups: severely negative (< –5000 mL, n = 4), moderately negative (–2000 to –5000 mL, n = 7), mildly negative (0 to –2000 mL, n = 14), mildly positive (0 to +2000 mL, n = 15), moderately positive (+2000 to +5000 mL, n = 16), and severely positive (> +5000 mL, n = 46).

• 178 patients were identified after the initial query.

• After initial data validation, the cohort was reduced to 158 patients.

• Following revision with the use of strict inclusion and exclusion criteria, including the removal of records with missing fluid balance data or invalid ICU stay records, the final cohort comprised 102 unique ICU admissions (Fig. **1**). Pulmonary arterial hypertension (PAH) is a rare condition, affecting an estimated 15 to 50 people per million in the general population. Because of its low prevalence, even a large critical care database like MIMIC-IV is expected to contain relatively few confirmed cases. After narrowing our search to include only patients with primary causes and excluding those with secondary causes, we identified a final cohort of 102 patients. While modest in size, this number is consistent with the expected rarity of the disease.

### 2.6 Variables

(1) **Demographics:** Age, gender.

(2) **Outcomes:** ICU mortality, LOS, ventilator use, and RRT needs. Mortality was defined as death occurring during the ICU admission window, using ICU ‘in time’ and ‘out time’ timestamps. ICU deaths occurring after ICU discharge were not included. RRT use was defined as any renal replacement therapy administered during the ICU stay. MIMIC-IV does not reliably distinguish between patients with chronic dialysis and those started de novo on RRT in the ICU.

(3) **Physiological Parameters:** Data required to calculate APACHE II scores, including mean arterial pressure, arterial pH, PaO2, and temperature.

(4) **Diagnoses:** Subgroup analysis of sepsis, pancreatitis, diabetic ketoacidosis (DKA), hyperosmolar hyperglycemic state (HHS), hypovolemic shock, and hemorrhagic shock.

(5) **Fluid Balance:** Cumulative fluid input, output, and net fluid balance during the ICU stay.

To facilitate stepwise analysis, patients were categorized into six fluid balance groups:

- Severely negative (< –5000 mL)
- Moderately negative (–2000 mL to –5000 mL)
- Mildly negative (0 mL to –2000 mL)
- Mildly positive (0 mL to +2000 mL)
- Moderately positive (+2000 mL to +5000 mL)
- Severely positive (> +5000 mL)

These ranges were selected to balance clinical interpretability with sample-size limitations, recognizing that narrower cutoffs would yield very small subgroups with limited statistical power.

### 2.7 Statistical Analysis

Model diagnostics included assessment of multi-collinearity and goodness-of-fit. Logistic regression results were validated using ridge regression with L2 regularization. Fluid balance subgroup definitions were consistently applied in regression models, and subgroup analyses were interpreted as exploratory due to limited sample sizes.

All analyses were conducted using Python and StatsModels. Descriptive statistics, including mean, standard deviation, and proportions, were used to summarize the cohort characteristics. Categorical variables were compared using the chi-square test or Fisher’s exact test as appropriate. Continuous variables were compared using Student’s t-test with unequal variance. Significance was defined as *p* < 0.05. Logistic regression was conducted to examine the relationship between fluid balance and outcomes, including ICU mortality, RRT use, ventilator use, and ICU LOS. Odds ratios with 95% confidence intervals were calculated to determine the strength and significance of these associations. Fluid balance subgroups, as defined above in the Variables section, were consistently applied in regression analyses. This study complies with the ethical requirements of using the MIMIC-IV database, which is de-identified and publicly available for research purposes. Access to the database required successful completion of the CITI “Data or Specimens Only Research” training program, ensuring proper handling of the dataset. No additional ethical approval was required.

## 3 RESULTS

This study investigated the relationship between fluid balance and key clinical outcomes in ICU patients with PAH, including ICU mortality, ventilator use, RRT use, and ICU LOS. The cohort was stratified into two groups based on fluid balance: negative fluid balance (n=25 patients) and positive fluid balance (n=77 patients) (Table **1**). The mean APACHE II score was 11.13 (SD 3.58), with no significant difference between fluid balance groups (*p* = 0.832). Baseline characteristics are summarized in Table **1**

**Fig. (2):**
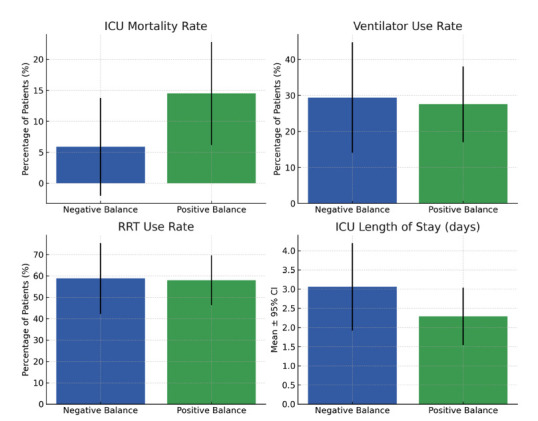
Clinical outcomes by fluid balance group with 95% confidence intervals. Patients were stratified into negative (n = 25) and positive (n = 77) fluid balance groups. Compared with the positive fluid balance group, the negative fluid balance group demonstrated a lower ICU mortality rate (5.9% *vs*. 14.5%). Ventilator use (29.4% *vs*. 27.5%) and renal replacement therapy (58.8% *vs*. 58.0%) were similar between groups. ICU length of stay was modestly longer in the negative balance group (3.06 ± 3.39 days *vs*. 2.29 ± 3.18 days). Bars represent group means or proportions, and error bars indicate 95% confidence intervals.

Patients with positive fluid balance had a higher ICU mortality rate (14.49%) than those with negative fluid balance (5.88%) (Table **1**). Ventilator use was similar between groups (27.54% *vs*. 29.41%), and RRT use remained comparable (57.97% *vs*. 58.82%). However, ICU LOS was slightly shorter in the positive fluid balance group (2.29 days) than in the negative fluid balance group (3.06 days) (Table **1** and Fig. **2**), possibly due to the higher ICU mortality. To better discern the potential threshold effects of fluid status on outcomes and identify an optimal range for management, we classified net fluid balance into distinct subgroups (*e.g.*, ‘severely positive,’ ‘mildly positive,’ *etc*.) to facilitate more granular comparisons across varying degrees of fluid overload or depletion Table **2**.

Patients with severely negative fluid balance (> -5000 mL) exhibited no ICU mortality but had high RRT use (83.3%) and longer ICU stays (4.67 days). In contrast, those with severely positive fluid balance (> +5000 mL) had the highest ICU mortality rate (19%) and the longest ICU LOS (6.16 days). Interestingly, patients with moderate negative to mildly positive fluid balance (−2000 mL to +2000 mL) had the lowest ICU mortality rates.

**Fig. (3):**
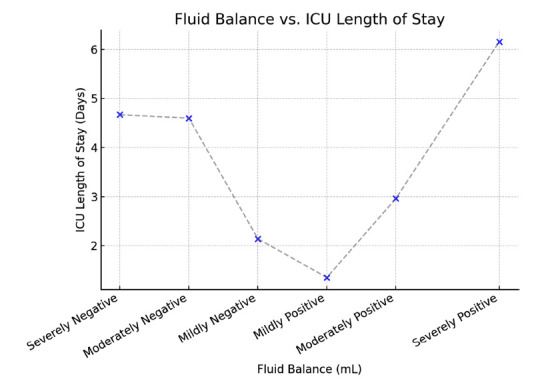
Relationship between fluid balance and ICU length of stay.

A scatter plot (Fig. **3**) illustrates the relationship between fluid balance and ICU LOS, suggesting that severely positive fluid balance is associated with prolonged ICU stays. Those with moderate negative fluid balance (~-2000 mL) to moderately positive fluid balance (~+2000 mL) had shorter ICU stays.

Univariate logistic regression analysis further evaluated these relationships Table **3**. Positive fluid balance was associated with a 74% increase in ICU mortality odds (OR: 1.74, 95% CI: 1.10–2.85). These findings are in line with earlier ICU studies showing that both positive and negative fluid balance can be linked to worse survival compared to more neutral states. Additionally, there was a trend toward higher ventilator use in the positive fluid balance group (OR: 2.11, 95% CI: 0.96–4.64), although this result did not reach statistical significance. Ridge regression Table **4** analysis supported an increased risk of ICU mortality in patients with positive fluid balance, with an OR of 1.62 [1.37-1.92].

Patients with negative fluid balance served as the baseline (reference) in the regression analysis. This means that the odds ratios (OR) for positive fluid balance are interpreted relative to the negative fluid balance group.

This scatter plot illustrates the relationship between fluid balance (in mL) and ICU length of stay (in days). Patients with severe positive fluid balance (>2000 mL) had prolonged ICU stays, whereas those with moderate negative fluid balance (~-2000 mL) had shorter ICU stays.

## 4 DISCUSSION

The findings of this study suggest that positive fluid balance in PAH patients is significantly associated with increased ICU mortality. Patients with positive fluid balance had an ICU mortality rate more than twice that of those with negative fluid balance (14.49% *vs*. 5.88%). In addition to these baseline statistics, the impact of positive fluid balance on ICU mortality was further highlighted through both univariate logistic regression and ridge regression analyses. In the ridge regression model, positive fluid balance was linked to 62% higher odds of ICU mortality (OR: 1.62 [1.37-1.92]), with the confidence interval excluding 1, indicating statistical significance. Similarly, univariate logistic regression showed that positive fluid balance was associated with 74% higher odds of mortality (OR: 1.74 [1.10-2.85]). The observed increase in mortality among patients with higher positive fluid balance correlates with previous studies suggesting that excessive fluid administration contributes to RV ischemia, worsening RV function, increased PVR, and reduced cardiac output, ultimately leading to right heart failure [17, 18].

Conversely, moderate negative fluid balance was associated with lower mortality rates and shorter ICU stays, supporting the hypothesis that judicious diuresis and controlled fluid removal strategies are beneficial in PAH patients (11). Specifically, patients with moderately negative to mildly positive fluid balance (–2000 mL to +2000 mL) had the lowest mortality and shortest ICU stays, highlighting a possible optimal fluid balance range. These results align with previous literature advocating for careful preload reduction in PAH, as higher right atrial pressures (>8 mmHg) correlate with increased mortality [19, 20]. Although no formal guidelines define fluid overload thresholds in PAH, we used stepwise net fluid ranges to evaluate potential dose-response effects. Also, there is currently no universally accepted definition of fluid overload in the ICU population [16]. Our categories allowed for clinically intuitive groupings while remaining transparent in methodology.

In addition to the direct effects of fluid overload on right ventricular (RV) function, it is important to consider that positive fluid balance may represent a surrogate marker for end-stage right heart failure or failure to respond to diuretic therapy, rather than a purely causal factor. Unfortunately, specific reasons for ICU admission (*e.g.*, decompensated RV failure *vs*. other acute triggers) were not consistently available in MIMIC-IV, which limited our ability to differentiate between therapeutic fluid administration, resuscitation requirements, and pathologic fluid accumulation.

The wide subgroup ranges (*e.g.*, –2000 mL to +2000 mL *vs*. ±5000 mL) were chosen to ensure sufficient patient counts for analysis. While narrower increments might better capture clinically distinct states (*e.g.*, a patient at –1500 mL *vs*. +1500 mL), further subdivision would have yielded groups too small for meaningful statistical comparisons. For this reason, our subgroup analyses should be considered exploratory, hypothesis-generating findings rather than definitive thresholds.

Our results align with existing pathophysiology: moderate negative to mildly positive balance (–2000 to +2000 mL) may reflect a compensated fluid state, whereas severely positive or negative balance likely indicates clinical instability.

### 4.1 Comparison to Literature on Hemodynamic Monitoring & Fluid Management

Prior studies emphasize that PAH patients experiencing acute decompensation require aggressive yet precise hemodynamic monitoring to prevent progression to RV failure and multiorgan dysfunction [8]. Though echocardiographic data were not available in the MIMIC-IV v3.1 database, the observed association between fluid balance and mortality may reflect hemodynamic stressors on an already compromised right ventricle. This remains speculative but aligns with known pathophysiologic mechanisms in PAH. Hoeper & Granton (2012) [3] recommend defining RV failure in PAH as a cardiac index <2.5 L/min/m^2^ and a right atrial pressure >8 mmHg, parameters that strongly correlate with poor prognosis. Additionally, studies highlight the importance of measuring PVR, central venous oxygen saturation (SvO2), and lactate levels to guide individualized fluid therapy [9, 13]. Fluid balance management in PAH patients is complex and highly individualized. Unlike left ventricular failure, if right ventricular failure ensues, it is preload-dependent, and excessive volume removal can paradoxically worsen cardiac output and systemic perfusion [10, 20]. At the same time, excessive volume can cause the failing RV to enlarge, resulting in septal bowing and worsening LV function [18]. Therefore, precise fluid titration is critical - reducing congestion while maintaining adequate intravascular volume.

Implementing advanced hemodynamic monitoring can offer real-time insights into right ventricular function and guide fluid management strategies [8]. Techniques such as pulmonary artery catheterization allow for direct measurement of pulmonary artery pressures and cardiac output, facilitating tailored interventions [8, 21]. Emerging technologies, including implantable hemodynamic monitors, have shown promise in providing continuous data, potentially improving patient outcomes [22].

The PAC-Man Trial (Pulmonary Artery Catheters in Management of Patients in Intensive Care) was a multicenter, randomized controlled study conducted to evaluate the impact of pulmonary artery catheter (PAC) use on hospital mortality among critically ill patients [23]. Although the trial did not demonstrate a mortality benefit with the routine use of pulmonary artery catheters (PACs) in the critical care setting, its findings may not universally apply, particularly in patients with severe PAH. In the context of advanced PAH and imminent right ventricular failure, real-time hemodynamic data from a PAC can be invaluable. Unlike other noninvasive methods that provide indirect estimates, PACs enable dynamic, moment-to-moment adjustments in therapy, potentially averting hemodynamic collapse. This precise hemodynamic monitoring is crucial for optimizing fluid management, titrating vasopressors, and guiding advanced therapies such as inotropic support [3]. The selective reintroduction of PAC use in complex cases of PAH possibly represents a nuanced, patient-centered approach that accounts for the unique pathophysiology and life-threatening risks inherent to this population. Moreover, concerns about complications associated with routine PAC placement in the ICU are likely to be substantially mitigated in the modern era, given the widespread adoption of ultra-sonography for procedural guidance [24].

### 4.2 Limitations & Future Research

Several limitations must be emphasized. First, the final cohort of 102 patients represents a 42% reduction from the 178 initially identified cases due to missing or incomplete data. This attrition introduces potential selection bias and limits the generalizability of our findings to the broader PAH population.

Second, although we stratified patients into fluid balance subgroups, several categories contained very few patients (*e.g.*, the severely negative group had only four patients). As such, subgroup comparisons should be interpreted cautiously and viewed as exploratory.

Third, we relied on cumulative fluid balance totals without considering timing, rate of administration, or clinical context. It is therefore unclear whether the positive balance reflected necessary resuscitation, iatrogenic administration, or pathologic accumulation. Future studies should integrate the temporal dynamics of fluid balance. Beyond this, future work could expand beyond traditional regression models by incorporating advanced computational approaches. For example, ensemble learning has shown promise in cardiovascular risk prediction [25, 26], while federated learning offers a framework for scaling analyses across multiple hospitals while preserving patient privacy [27]. Such methods may allow validation of our findings in larger, more diverse PAH populations.

Fourth, we excluded patients with comorbidities and secondary forms of pulmonary hypertension. While this allowed us to focus on idiopathic PAH, it may not fully capture the modern phenotype of PAH patients, who are increasingly older and carry multiple comorbidities. This limits external validity.

Finally, diuretic treatment, hemodynamic parameters (*e.g.*, right atrial pressure, cardiac index), and echocardiographic data were not reliably available in MIMIC-IV, preventing more nuanced interpretation of the fluid balance–outcome relationship.

## CONCLUSION

This study explored the relationship between fluid balance and ICU outcomes in patients with PAH. Our findings suggest that positive fluid balance may be associated with increased ICU mortality and prolonged ICU stays, highlighting how volume status may affect ICU outcomes in critically ill PAH patients. Conversely, moderate negative fluid balance appeared to be associated with improved outcomes, suggesting that careful fluid management could help optimize ICU care for this population. Based on the results, we postulate that an ideal fluid balance may range from– 2L to +2L.

Individualized, hemodynamic-guided fluid management strategies are needed in PAH patients admitted to the ICU. While fluid overload may contribute to worse outcomes, excessive volume removal could also pose risks, necessitating a balanced, tailored approach. Given the retrospective nature and limited sample size of this study, further research is needed to better define optimal fluid management strategies and their impact on PAH-related outcomes.

## Figures and Tables

**Table 1:** Baseline characteristics of study population by fluid balance group.

Metric	Negative Group	Positive Group	*p*-value
Sample Size	25	77	—
ICU mortality Rate (%)	5.88%	14.49%	0.221
Ventilator Use (%)	29.41%	27.54%	1.000
RRT Use (%)	58.82%	57.97%	0.944
ICU LOS (days)	3.06 ± 3.39	2.29 ± 3.18	0.130
APACHE II Score	11.00 ± 3.28	11.19 ± 3.69	0.832
Age (years)	59.7 ± 15.2	57.9 ± 14.3	0.620
Female (%)	50.00%	47.83%	0.845

**Table 2:** Summary of fluid balance subgroups and associated clinical outcomes.

Fluid Balance Subgroup	Patient Count (%)	ICU Mortality Rate	RRT Count	Ventilator Count	Avg ICU LOS (Days)
Severely negative	4 (3.88%)	0	5	1	4.67
Moderately negative	7 (6.8%)	0	5	5	4.6
Mildly negative	14(13.59%)	0.1	13	5	2.14
Mildly positive	15(14.56%)	0.13	14	4	1.35
Moderately positive	16(15.53%)	0.16	13	8	2.96
Severely positive	46(44.66%)	0.19	42	45	6.16

**Note:** Severely negative: > -5000mL

Moderately negative: -2000mL to –5000mL

Mildly negative: 0mL to -2000mL

Mildly positive: 0mL to 2000mL

Moderately positive: 2000mL to 5000mLSeverely positive: > 5000mL

**Table 3:** Univariate logistic regression analysis of fluid balance and clinical outcomes.

Outcome	Odds Ratio [95% CI]	Interpretation
ICU mortality	1.74 [1.10-2.85]	Positive fluid balance is associated with 74% higher odds of ICU mortality.
Ventilator needs	2.11 [0.96-4.64]	Higher odds of ventilator needs for positive fluid balance, but not statistically significant.
CRRT needs	0.81 [0.38-1.72]	Lower odds of CRRT need for positive fluid balance. Not statistically significant.
ICU LOS (days)	1.37 [-0.66-3.40]	Longer ICU stays for positive fluid balance. Not statistically significant.

**Table 4:** Interpretation of ridge logistic regression results (adjusted for APACHE II score).

Outcome	Odds Ratio [95% CI]	Interpretation
ICU mortality	1.62 [1.37-1.92]	Positive fluid balance is associated with 62% higher odds of ICU mortality. CI does not include 1, suggesting statistical significance.
Ventilator needs	1.36 [0.96-1.91]	Higher odds of ventilator needs for positive fluid balance, but CI includes 1, suggesting borderline significance.
CRRT needs	0.91 [0.7-1.19]	No significant association between positive fluid balance and CRRT needs.
ICU LOS (days)	0.58 [-0.72-1.87]	Negative coefficient suggests shorter ICU LOS, but CI includes 0, meaning it's not statistically significant.

**Note:** Ridge regression was applied instead of standard multivariate logistic regression to prevent overfitting and handle multicollinearity between predictors. Traditional multivariate logistic regression may produce unstable estimates in such cases, as correlated predictors can lead to inflated coefficients. By applying L2 regularization, ridge regression shrinks coefficients and improves generalizability, making it a more reliable approach.

## LIST OF ABBREVIATIONS

PAH: Pulmonary arterial hypertension
PVR: Pulmonary vascular resistanc
IPAH: Idiopathic pulmonary arterial hyperte

## STANDARDS OF REPORTING

STROBE guidelines were followed.
